# SURVEILLANCE AND MONITORING: Toxics Report Improves, but Data Still Limited

**DOI:** 10.1289/ehp.117-a390

**Published:** 2009-09

**Authors:** Bob Weinhold

**Affiliations:** **Bob Weinhold**, MA, has covered environmental health issues for numerous outlets since 1996. He is a member of the Society of Environmental Journalists

One of the gaping holes in the public health tool kit is the lack of comprehensive knowledge about the occurrence and toxic effects of the full spectrum of chemicals emitted by industrial facilities. The Commission for Environmental Cooperation (CEC) has taken a small step toward filling this void by assembling selected basic toxics data for North America with the 10 June 2009 release of its 12th annual report, *Taking Stock: 2005 North American Pollutant Releases and Transfers*. The CEC oversees the North American Agreement on Environmental Cooperation, which supports the environmental provisions of the North American Free Trade Agreement.

*Taking Stock* pulls together the latest data available for all 3 countries on industrial emissions that are tracked under categories such as toxic releases and transfers, criteria air pollutants, fugitive emissions, and greenhouse gases. The report applies a single risk-scoring metric, the toxic equivalency potential (TEP), to make an apples-to-apples comparison of the relative potential threat posed by some of the toxics tracked. And it focuses in particular on North American petroleum industry pollutants, providing what the CEC says is the most complete reporting yet on this industrial sector.

The report draws much of its data from each country’s pollutant release and transfer register (PRTR): the Toxics Release Inventory (TRI) of the United States, the National Pollutant Release Inventory (NPRI) of Canada, and the Registro de Emisiones y Transferencia de Contaminantes (RETC) of Mexico. PRTRs examine the media into which pollutants are released—air, water, land, and underground injection—along with transfers of pollutants offsite for recycling, energy recovery, treatment, or other management. Criteria air pollutants are tracked separately, and greenhouse gases are identified through a third data set.

From a very basic perspective, the *Taking Stock* report series is favorably received. “It does help to see all the data together, using all the same units of measurement,” says Richard Valentinetti, director of the Air Pollution Control Division within the Vermont Department of Environmental Conservation. He also notes that it’s important to monitor and address pollution problems across borders.

But he believes the CEC reports provide limited overall value. “There always are problems comparing data between the three countries,” he says. That’s because the countries’ tracking systems for various pollutants have widely divergent reporting requirements that won’t be synchronized anytime soon. It’s also due to underlying weaknesses of each individual database. “In the United States, we’re still not doing a good job with normal emission inventories,” he says.

Even with such limitations—which the CEC acknowledges—the report is useful for identifying and examining large-scale, continent-wide problems, says François Lavallée, manager of Environment Canada’s Comprehensive Inventory Compilation and Quality Assurance/Quality Control Section. He anticipates that future reports will become increasingly beneficial since this is just the second year that data for Mexico have been included.

The available data show that the continent’s air, water, surface, and subsurface received at least 8,484 billion kg of greenhouse gases, 32 billion kg of criteria air pollutants, and 5.5 billion kg of potentially toxic releases and transfers in 2005. The United States was the primary source, in part because it hosted 82% of the 35,023 industrial facilities required to report at least 1 of the pollutants (of about 889,000 such facilities continent-wide). Canada hosted 12% of the reporting facilities, and Mexico 6%. Because facility-specific data on greenhouse gases are not widely available, the report provides only an overview of these pollutants, which are also produced by non–point sources such as vehicles, agriculture, wildfire emissions, and commercial and residential properties—sources not subject to PRTR reporting but sometimes included in criteria air pollutant or greenhouse gas inventories.

For substances tracked by the PRTRs, the top overall emitters in Canada were oil and gas extraction, primary metal manufacturing (e.g., smelters), and publicly owned wastewater treatment plants. In Mexico, the leaders were metal mines, electric utilities, and electrical equipment manufacturing. In the United States, chemicals manufacturing, primary metal manufacturing, and mines and quarries led the way. In all 3 countries, large quantities of releases and transfers were reported for the chemicals manufacturing and transportation equipment manufacturing sectors. However, inconsistent reporting requirements—including nomenclature differences—preclude continent-wide comparisons of industries.

TEP calculations were derived through a method developed at the University of California, Berkeley, that expresses a chemical’s developmental/reproductive toxicity and carcinogenicity in terms of comparable amounts of toluene and benzene, respectively. The Berkeley method is just one of many such methods, each of which can lead to very different risk findings. TEP calculations in this report apply only to air and water releases and do not provide calculations for other health end points, such as respiratory, cardiovascular, neurologic, or immunologic damage.

Of the substances reported, the CEC determined that mercury and its compounds posed by far the greatest potential health threat, with a TEP for developmental/reproductive risk equivalent to 975.2 billion kg for air releases and 187.5 billion kg for water releases. Next in terms of potential developmental/reproductive toxicity were lead and its compounds, copper and its compounds, arsenic and its compounds, and hydrochloric acid. In addition to its reproductive/developmental effects, air releases of arsenic and its compounds topped the list of carcinogens with a TEP risk equivalent of 947.0 million kg, along with 313.0 million kg for water releases. Other leading carcinogens included chromium and its compounds, lead and its compounds, glycol ethers, and hydrogen sulfide. There are efforts under way to improve the data used in the report.

However, by grouping metals with their related compounds, some of which may be more or less toxic than the parent metal—as in the case of chromium and arsenic—it is possible the report may miscalculate the risk posed by the total amount emitted. Mexican officials are reviewing health and toxicity data for some pollutants, says Orlando Cabrera-Rivera, the CEC’s program manager for air quality and PRTR. That could lead to more substances being added to the 104 currently on Mexico’s RETC (in comparison, the NPRI currently lists 323 substances, and the TRI lists 600). Cabrera-Rivera also says that, in addition to facilities under federal jurisdiction, Mexican officials have been adding reporting requirements for some facilities under state jurisdiction. The countries also are cooperating on developing sector profiles in order to establish baselines and thus improve the quality of the data.

Lavallée says Canada has worked at making its PRTR mesh fairly well with that of the United States, but he says it’s unlikely his country will add many more substances solely to increase comparability. For instance, he says, many pesticides are listed on the TRI but not on Canada’s NPRI because the Canadian pesticide manufacturing industry is only about one-tenth the size of its U.S. counterpart, making emissions from this sector a relatively low Canadian priority. Most changes to the NPRI will be driven by Canada’s Chemicals Management Plan and Clean Air Regulatory Agenda. Data on the greenhouse gas categories addressed by the CEC could also improve as reporting requirements in the United States kick in.

Tracking all the substances in this year’s report captures less than 0.5% of the 239,000 substances that are regulated or included in inventories worldwide and just 3% of the 30,000 chemicals that are most widely used commercially in Canada and the United States. However, points out Cabrera-Rivera, “While it would be important to include some other pollutants on the PRTR lists, this should be done by prioritizing based on sector pollutant profiles, as well as the potential risk posed by pollutants of concern, since all pollutants are not equal.”

Identifying all toxics in the environment, their by-products, and their adverse health effects remains a daunting challenge. But the obstacles to be overcome are disarmingly simple: “Time, resources, and quality of data,” Valentinetti says.

## Figures and Tables

**Figure:**
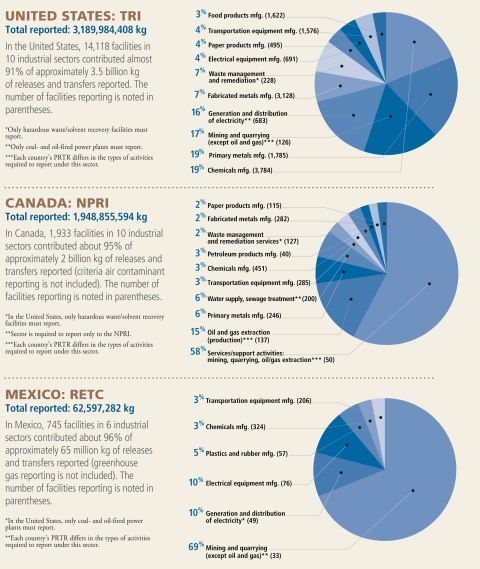
**Largest Releases/Transfers** by Industry in North America

**Table:** **Top 30 Pollutants** in North America (2005)

Pollutant		Mandatory Reporting			Toxicity	Released (%)	Transferred (%)	Total (kg)
		CA	MX	US		Percentages may not sum to 100% due to rounding		
1	Hydrogen sulfide	•	•	•		39	61	1,368,487,605
2	Zinc and its compounds	•		•	P	64	36	639,516,966
3	Lead and its compounds	•	•	•	DCP	58	42	453,766,645
4	Copper and its compounds	•		•	P	20	79	422,509,715
5	Nitrate compounds	•		•		72	27	261,638,682
6	Hydrochloric acid	•		•		99	1	259,799,720
7	Methanol	•		•		48	51	235,476,261
8	Manganese and its compounds	•		•		60	41	196,817,633
9	Ammonia	•		•		93	7	168,527,542
10	Sulfuric acid	•		•		54	45	166,764,975
11	Barium and its compounds			•		99	1	111,360,662
12	Toluene	•		•	D	27	74	101,536,968
13	Arsenic and its compounds	•	•	•	DCP	99	0	90,986,426
14	Chromium and its compounds	•	•	•	CP	33	67	87,902,059
15	Nickel and its compounds	•	•	•	DCP	24	76	77,413,728
16	Xylenes	•		•		23	78	76,951,478
17	Ethylene glycol	•		•		5	94	54,799,080
18	Hydrogen fluoride	•		•		95	4	36,115,698
19	Styrene	•	•	•	C	70	30	35,196,460
20	*n*-Hexane	•		•		52	48	33,592,714
21	Vanadium and its compounds	•		•	C	84	16	30,587,841
22	Dichloromethane	•	•	•	C	9	90	30,234,488
23	Aluminum (fume or dust)	•		•		77	24	27,413,799
24	Phosphorus	•		•		78	22	27,213,543
25	Ethylene	•		•		43	58	21,481,170
26	Glycol ethers			•		47	54	21,239,844
27	*n*-Butyl alcohol	•		•		42	58	18,289,635
28	Asbestos (friable form)	•	•	•	C	99	0	310,703
29	1,2-Dichloroethane	•	•	•	C	0	100	221,011
30	Formaldehyde	•	•	•	C	100	0	158,162

**Note:** C = carcinogen; D = developmental/reproductive toxicant; P = persistent bioaccumulative toxicant.

Adapted from CEC. 2009. Taking stock: 2005 North American pollutant releases and transfers. Montréal: Commission for Environmental Cooperation; p. 41.

